# 0997. The unrecognized effects of phosphodiesterase 4 on epithelial cells in pulmonary inflammation

**DOI:** 10.1186/2197-425X-2-S1-P82

**Published:** 2014-09-26

**Authors:** F Konrad, A Bury, MA Schick, K-C Ngamsri, I Vollmer, J Reutershan

**Affiliations:** University of Tübingen, Tübingen, Germany; University of Würzburg, Würzburg, Germany

## Introduction

Acute pulmonary inflammation (ALI) is characterized by migration of polymorphonuclear neutrophils (PMNs) into the different compartments of the lung, passing an endothelial and epithelial barrier. Recent studies showed evidence that phosphodiesterase (PDE)4-inhibitors stabilized endothelial cells [1]. PDE4B and PDE4D play a pivotal role in inflammation, whereas blocking PDE4D is suspected to cause gastrointestinal side effects.

## Objectives

To investigate the particular role of PDE4 on PMN migration and lung epithelium.

## Methods

ALI was induced by inhalation of LPS in mice. PDE4-inhibitors roflumilast and rolipram were administered i.p. or were nebulized after inflammation to investigate the impact of PDE-inhibitors on pulmonary epithelium in vivo. Additional, PDE4-inhibitors were tested regarding to migration of human PMNs through pulmonal epithelial barrier in vitro. Furthermore the influence of PDE4-inhibitors on PDE4B and PDE4D expression as well as on cytokines were analyzed.

## Results

Both PDE4-inhibitors decreased transendothelial and transepithelial migration, whereas roflumilast showed a superior effect compared to rolipram on the epithelium. Both inhibitors decreased the chemokines TNFα, IL6, and CXCL2/3. CXCL1, the strong PMN chemoattractant secreted by the epithelium [2], was significantly more reduced by roflumilast compared to rolipram. In vitro assays with human PMNs and human pulmonary epithelium also emphasized the pivotal role of roflumilast on the epithelium. Expression of PDE4 subtypes B and D were both increased in the lungs by LPS, and PDE4 inhibitors decreased mainly PDE4B. Administration of the PDE-inhibitors still had anti-inflammatory properties after LPS inhalation and the topical administration of PDE4-inhibitors was also effective in curbing down PMN migration, both highlighting the clinical potential of these compounds.

## Conclusions

We determined the pivotal role of the PDE4-inhibitor roflumilast on lung epithelium and emphasized its main effect on PDE4B in hyperinflammation.

## References

[1] Schick MA, Wunder C, Wollborn J, Roewer N, Waschke J, Germer CT, Schlegel N (2012). Phosphodiesterase-4 inhibition as a therapeutic approach to treat capillary leakage in systemic inflammation. J. Physiol.

[2] Becker S, Quay J, Koren HS, Haskill JS (1994). Constitutive and stimulated MCP-1, GRO alpha, beta, and gamma expression in human airway epithelium and bronchoalveolar macrophages. Am. J. Physiol.

